# Research progress on ferroptosis in colorectal cancer

**DOI:** 10.3389/fimmu.2024.1462505

**Published:** 2024-09-18

**Authors:** Yuan Li, Yao Bi, Wenjing Li, Yingshi Piao, Junjie Piao, Tong Wang, Xiangshan Ren

**Affiliations:** ^1^ Central Laboratory, Yanbian University Hospital & Key Laboratory of Pathobiology, Yanbian University, State Ethnic Affairs Commission, Yanbian University, Yanji, China; ^2^ Department of Pathology & Cancer Research Center, Yanbian University, Yanji, China; ^3^ Department of Anesthesia, Yanbian University Hospital, Yanji, China; ^4^ Department of Gynecology, Yanbian University Hospital, Yanji, China; ^5^ Key Laboratory of Natural Medicines of the Changbai Mountain, Ministry of Education, Yanbian University, Yanji, China

**Keywords:** colorectal cancer, ferroptosis, regulation, immune cell, treatment

## Abstract

Ferroptosis is a new form of cell death that differs from traditional forms of death. It is ferroptosis-dependent lipid peroxidation death. Colorectal cancer(CRC) is the most common tumor in the gastrointestinal tract with a long occultation period and a poor five-year prognosis. Exploring effective systemic treatments for CRC remains a great challenge worldwide. Numerous studies have demonstrated that ferroptosis can participate in the biological malignant process of various tumor, including CRC, so understanding the role and regulatory mechanisms of ferroptosis in CRC plays a crucial role in the treatment of CRC. In this paper, we reviews the mechanisms of ferroptosis in CRC, the associated regulatory factors and their interactions with various immune cells in the immune microenvironment. In addition, targeting ferroptosis has emerged as an encouraging strategy for CRC treatment. Finally, to inform subsequent research and clinical diagnosis and treatment, we review therapeutic approaches to CRC radiotherapy, immunotherapy, and herbal therapy targeting ferroptosis.

## 1 Introduction

Colorectal cancer (CRC) is the third leading cause of cancer-related death globally and the third most prevalent cancer worldwide (1, 2). Over the past few decades, the incidence of CRC has either dropped or been consistent in high-income nations; nevertheless, the incidence of CRC detected in younger people, or early-onset colorectal cancer, has been rising globally (3, 4). Due to its asymptomatic, highly invasive, intra - and intertumor heterogeneity, and resistance to chemotherapy, treatment options for CRC are limited, resulting in poor overall prognosis and survival rates. A study from China suggests an optimal interval of 10 weeks for totally total mesorectal excision (TME) after neoadjuvant chemoradiotherapy (CRT) to improve recurrence-free survival (RFS) in patients with locally advanced colorectal cancer (5). Despite notable advancements in screening, diagnosis, surgery, and systemic therapy, the five-year overall survival rates are still low, hovering around 10%-15% (6). Therefore, it is crucial to discover new treatments and effective drugs that have low toxicity and minimal side effects for CRC patients.

Ferroptosis is a novel type of regulatory cell death(RCD) that was first proposed by Dixon in 2012 (7). In recent years, ferroptosis has been linked to several cancers, where it has affected both the course of the disease and the response to treatment. Ferroptosis inducers inhibit the proliferation of a variety of cancers and work synergistically with traditional drugs. For example, the ferroptosis inducer erastin inhibits head and neck cancer (HNC) cell growth and the accumulation of lipid reactive oxygen species, thereby inducing ferroptosis. And enhance cisplatin cytotoxicity of resistant HNC cells (8). Specific inhibition of ferroptosis defense mechanisms and inducing ferroptosis in tumor cells has emerged as an innovative approach for cancer treatment.

In treating tumors, many studies have found that inhibition of the metabolic processes involved in the ferroptosis pathway can reduce resistance to chemotherapeutic agents in tumor patients. In CRC cells, a variety of modulators have been shown to either stimulate or inhibit ferroptosis. However, damage caused by ferroptotic processes might result in immunosuppression linked with inflammation within the tumor microenvironment, which in turn promotes tumor growth. Therefore, due to the complexity of the tumor microenvironment (TME), no real clinical benefit has been obtained. For cancer, there is a complicated interaction between TME cells and ferroptosis. Ferroptosis, as an important mode of tumor cell death, is associated with tumor infiltrating immune cells that exist in multiple connections. This review elucidates the role of ferroptosis in CRC and its role in the TME and provides a theoretical foundation for the combined application of ferroptosis in CRC therapy.

## 2 Mechanism of ferroptosis and its regulation in CRC

Ferroptosis, unlike other types of RCD, is an iron-dependent and peroxide-driven form of death (9). The characteristics of ferroptosis include rupture of the outer mitochondrial membrane, vesiculation and rupture of the cell membrane, smaller mitochondria, higher membrane density, diminished or nonexistent mitochondrial ridges, and a normal-sized nucleus without chromatin condensation. Ferroptosis is a form of cell death caused by the excessive accumulation of lipid peroxides due to disturbances in intracellular metabolic pathways, and intracellular iron metabolism and lipid homeostasis are closely related.

### 2.1 Amino acid metabolism

**Figure 1:**
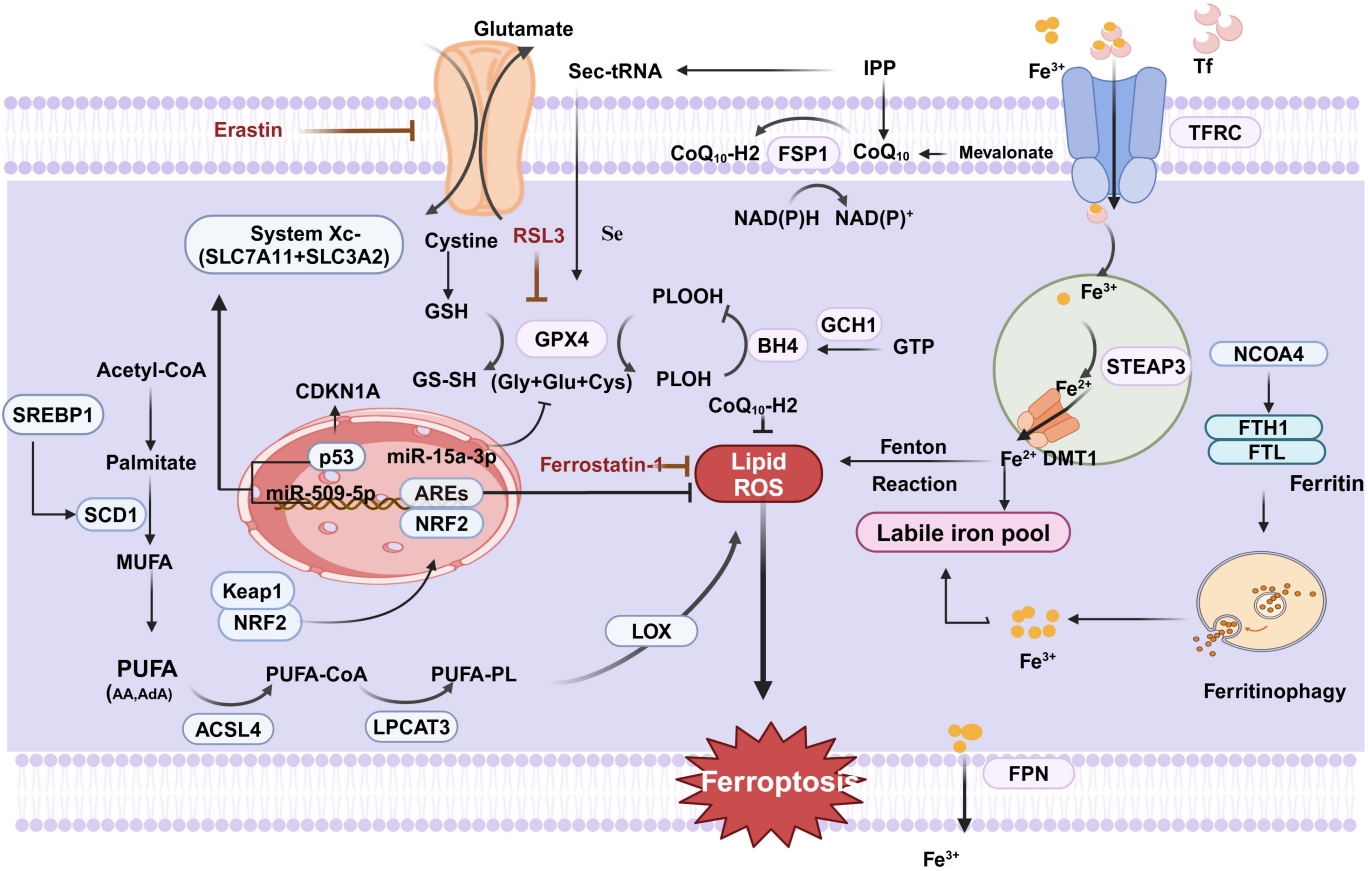
Regulation of ferroptosis in CRC. Cystine is transported by system XC- into the cell to participate in the synthesis of GSH, which can be oxidized to GSSH by GPX4, which converts PLOOH to PLOH and inhibits ferroptosis. ACSL4 catalyzes the ligation of PUFA and CoA to generate PUFA-CoA, followed by PUFA-PL catalyzed by LPCAT3, and finally lipid ROS catalyzed by LOX, which promotes ferroptosis. Finally, it generates lipid ROS catalyzed by LOX,which promotes cellular ferroptosis. In addition, extracellular Fe^3+^ enters the cell under the transport of TFR, and then is reduced to Fe^2+^ to form an unstable iron pool via STEAP3 and DMT1. Excess intracellular Fe^2+^ generates ROS through the Fenton reaction leading to the occurrence of ferric netting in cells.In addition FSP1 is a nicotinamide adenine dinucleotidase (NADP-) dependent coenzyme Q (CoQ) oxidoreductase. In addition, FSP1 relies on NADP- to reduce CoQ to CoQ10-H2,which inhibits lipid peroxidation and suppresses cellular ferroptosis. (Created with BioRender.com).

The two amino acid molecules primarily involved in regulating ferroptosis are glutathione peroxidase 4 (GPX4) and System Xc-. System Xc-, an inverse transporter protein located on the plasma membrane, consists of a light chain subunit solute carrier family 7 member 11(SLC7A11; also known as xCT)) and a heavy chain subunit solute carrier family 3 member 2 (SLC3A2) linked by a covalent disulfide bond. SLC7A11 is a multichannel protein responsible for amino acid transport, highly specialized for cystine and glutamate, and promotes glutathione (GSH) synthesis. The chaperone protein SLC3A2 keeps the SLC7A11 protein stable and location in the membrane (**Figure 1**). It has been shown that p53 may promote ferroptosis by either upregulating the expression of SAT1 and GLS2 or suppressing the expression of SLC7A11, but at the same time p53 can inhibit ferroptosis by suppressing DPP4 activity or inducing CDKN1A/p21 expression. System Xc- plays a crucial role in the cellular uptake of cystine and the maintenance of intracellular GSH levels (10, 11). GSH, a tripeptide made up of glutamate, cysteine, and glycine, is the main antioxidant agent of the antioxidant system in the body. The majority of intracellular GSH is present in reduced form, and it can oxidize to glutathione disulfide (GSSH) when it reacts with oxidizing agents like reactive oxygen species(ROS). GPX4 can maintain membrane lipid bilayer homeostasis by reducing lipid peroxides to normal phospholipid molecules using GSH as a substrate.

GPX4 is a key regulator of ferroptosis, the only enzyme in the cell that converts lipid peroxides to lipids. Therefore, the synthesis of GSH can be influenced by blocking system Xc- activity, leading to reduced GPX4 activity and decreased antioxidant capability of the cell, ultimately resulting in ferroptosis (12, 13). For instance, the expression of both miR-509-5p and miR-15a-3p was significantly decreased in CRC cells compared to normal colorectal cells. It was revealed that miR-509-5p and miR-15a-3p overexpression inhibited SLC7A11 and GPX4, key proteins for ferroptosis, respectively, to promote ferroptosis in CRC cells (14, 15). APC serves as a CRC oncogene, and 80% of sporadic CRC have mutations in the APC gene, which has been reported to be involved in IFNγ/IFNGR2/APC/TCF4/GPX4 axis-mediated organoid damage and to inhibit the efficacy of CRC stem cells (CSCs).It has been demonstrated that Eastin and RSL3 can trigger ferroptosis in various CRC cells by inhibiting System Xc- and GPX4 activity, respectively (16). In addition, Erastin stimulates ferroptosis in CRC resistant cells and reverses CRC resistance. In addition, it was found that knockdown of tp53-induced regulator of glycolysis and apoptosis (TIGAR) significantly increased ferroptosis in SW620 and HCT116 cells, suggesting that TIGAR is a potential regulator of ferroptosis resistance in CRC development. On the one hand, down-regulation of TIGAR significantly decreases the GSH/GSSG ratio, increases the production of lipid peroxidation products, and promotes the accumulation of the lipid peroxidation product malondialdehyde (MDA), which sensitized CRC cells to ferroptosis disease induced by erastin. On the other hand, stearoyl-CoA desaturase-1(SCD1) expression is suppressed by TIGAR inhibition in a redox- and AMPK-dependent manner (17).

### 2.2 Lipid metabolism

The body’s metabolism is significantly influenced by oxygen radical reactions and lipid peroxidation reactions, which are normally in a dynamic and coordinated equilibrium. Polyunsaturated fatty acids (PUFA) in lipids, especially arachidonic acid(AA), are susceptible to peroxidation reactions leading to cell membrane and organelle membrane instability and permeability, which eventually lead to cell death (14). The enzymatic reaction of lipid peroxidation is mediated by various factors such as acyl coenzyme A synthase long chain family member 4 (ACSL4), lysophosphatidylcholine acyltransferase 3 (LPCAT3), and lipoxygenases (LOXs). ACSL4 and LPCAT3 were the first genes to be found to promote ferroptosis because of their role in promoting PUFA doping of membrane lipids. ACSL4 is a lipid metabolizing enzyme required for lipid peroxidation. In the eukaryotic cytoplasmic matrix, ACSL4 activates PUFAs to form long-chain acyl CoA, which in turn is inserted into the phospholipids of the plasma membrane by LPCAT3. LOXs are non-heme iron-containing dioxygenases. As a distinct GPX4 regulator that stimulates membrane oxidative stress and GSH depletion, 12/15-LOX plays a role in the build-up of lipid peroxides during ferroptosis (18). LOX can catalyze the dioxygenation of PUFAs containing at least two independent cis-double bonds and oxidizes PUFAs to their corresponding hydroperoxy derivatives for further conversion into bioactive lipid mediators (19). All things considered, PUFA oxidation via ACSL4, LPCTA3, and LOX can induce ferroptosis and govern lipid peroxidation. What is more, we do not know how the ferroptosis-induced death pathway ultimately leads to cell death. The most downstream step identified so far is the uncontrolled peroxidation of PUFA-PLs, where peroxidized phospholipids have the potential to cause membrane damage or even membrane perforation, thereby compromising membrane integrity. However, more mechanisms remain to be investigated.

In conclusion, the intracellular redox state affects the sensitivity of cells to ferroptosis. The transcription factor nuclear factor E2-related factor 2 (Nrf2), which binds to antioxidant response elements (AREs) found in the promoter regions of numerous cytoprotective genes, is crucial for the oxidative stress response. In the physiological state, Nrf2 leads to proteasomal degradation by conjugating to kelch-like ECH-associated protein1(Keap1) in the cytoplasm. During cellular stress, the two dissociate, Keap1 releases Nrf2, and free Nrf2 binds to the ARE to drive antioxidant gene expression. As expected, it was demonstrated that Nrf2 inhibits cellular ferroptosis by blocking lipid peroxidation through the antioxidant system (20). Several drugs targeting Nrf2 have been shown to induce ferroptosis in CRC. In addition, there are new studies explaining the new mechanism of cetuximab treatment for KRAS, that is, cetuximab blocked the activation of Nrf2/HO-1 in RSL3-treated KRAS-mutant CRC cells, which increased RSL3-induced lipid ROS and MDA levels contributing to ferroptosis (21). Tagitinin C may cause ferroptosis by activating the PERK-Nrf2-HO-1 signaling pathway through endoplasmic reticulum stress (22). It has been shown that the combination of aspirin and the ferroptosis inducer RSL3 may be more effective in treating CRC harboring oncogenic PIK3CA mutations. This is because aspirin inhibits the PI3K/AKT/mTOR pathway, thereby promoting ferroptosis in CRC cells by inhibiting downstream SREBP-1/SCD1-mediated adipogenesis.

### 2.3 Iron metabolism

Ferroptosis, as its name implies, is caused by abnormal iron metabolism. Iron in cells is able to participate in functions such as basic energy metabolism, mitochondrial function and DNA synthesis within the cell. Dysregulation of intracellular iron homeostasis has been associated with a range of tumors, particularly CRC. CRC is the only malignancy that maintains two sources of iron uptake (systemic uptake of circulating iron and intestinal luminal iron absorption). The presence of excess iron in the gastrointestinal tract may promote proliferation and tumor transformation of colonocytes. Iron has two oxidation states, Fe^2+^ and Fe^3+^. If the source of iron is heme iron, it can be absorbed directly into cells via haem carrier protein 1(HCP1). However, if the source of iron is food, Fe^3+^ in the food needs to be reduced to Fe^2+^ first, and then absorbed into the small intestinal epithelial cells under the action of divalent metal transporter 1 (DMT1) (23, 24). A portion of the Fe^2+^ absorbed by the above pathways is stored as ferritin in the liver small intestine and macrophages for utilization by the organism when needed. The other part enters the circulation through ferroportin(FPN) on the outer side of the basement membrane and is oxidized to Fe^3+^, which in turn binds to transferrin in the blood and is transported to the target organ via the blood circulation in the form of Tf-Fe^3+^. Hepcidin produced by the liver binds to FPN to inhibit the transport of Fe3+, causing them to be sequestered in the cell. Fe^3+^ binds to the cell membrane’s transferrin receptor (TFRC/TFR1) and is reduced to Fe^2+^ by the metal reductase STEAP family member 3(STEAP3). DMT1 then releases Fe^2+^ into the cytoplasm’s labile iron pool. This fraction of Fe^2 +^ forms an labile iron pool that generates ROS leading to oxidative damage and promotes cellular ferroptosis, with most of the remaining iron synthesized into iron-binding proteins or stored in ferritin, which serves as a non-toxic, stable pool of iron that maintains the normal metabolic needs of the body. It is worth noting that FPN is uniquely required for the transport of Fe^3+^ from the cell to the blood, so the hepcidin-FPN axis is central to the modulation of iron homeostasis (21). Additionally, Andrew and colleagues provide experimental evidence that the hepcidin/FPN signaling axis, which regulates carcinogenesis and cancer progression, is essential to local iron management in CRC in colonic epithelial cells. In addition, nuclear receptor coactivator (NCOA4), a protein that mediates ferritin autophagy, can specifically recognize and mediate the degradation of ferritin heavy chain 1(FTH1) and Ferritin light chain(FTL) to lysosomes, increases intracellular Fe^2+^ levels and lipid peroxidation levels and promotes cellular ferroptosis (25). Iron homeostasis is preserved under normal physiological conditions via the dynamic iron pool. However, ferroptosis occurs when the body’s iron Fe^3+^ increases, and Fe^3+^ metabolism in the body can trigger oxidative stress, which can trigger ferroptosis by generating ROS through the Fenton reaction and activating iron-containing enzymes (e.g., LOX) to promote lipid peroxidation. A recent study showed that the transcription factor EB(TFEB), as a major regulator of the autophagy lysosomal pathway, maintains low levels of intracellular unstable iron by up-regulating the expression of FTL and FTH1, but this process may depend on TFR1-mediated lysosomal iron uptake (26). In a way, the dynamic equilibrium between stable and unstable iron pools is crucial for the regulation of cellular ferroptosis. Therefore, in recent years, there has been an increasing number of studies targeting iron ions for cancer treatment. RAS mutations are common in CRC and are hard to therapy. Previous research has demonstrated that KRAS may control JAK-STAT and HIF2α signaling, which in turn controls iron input mediated by DMT1, maintaining high iron levels necessary for CRC growth. Interestingly, there are findings that tumors in KRAS-mutant male CRV patients suppress ferroptosis, and that changes in gene expression that suppress ferroptosis correlate with poor outcomes in these patients. It has been demonstrated that the iron metabolism regulator iron responsive element binding protein 2 gene (IREB2) increases cellular sensitivity to ferroptosis and facilitates iron absorption. Research has demonstrated that through controlling iron metabolism, TF and IREB2 both increase the susceptibility of cells to ferroptosis. Moreover, it is reported that OTUD1, an immune activator with the function of regulating iron ion metabolism, promotes the immune clearance of tumor cells by inducing ferroptosis (27). FTH1 is the predominant ferritin in cells, which possesses Fe^2+^ oxidase activity and prevents oxidative damage induced by Fe^2+^-mediated Fenton reaction. Yujin et al. found studies showing a strong correlation between poor patient prognosis and high expression of ribosomal L1 structural domain containing 1 (RSL1D1) in CRC tissues. Knockdown of RSL1D1 down-regulated FTH1, which in turn increased the intracellular iron concentration and induced ferroptosis in CRC (28). Interestingly, in Parkinson’s disease, iron plays an important role in the process of cell senescence induced by α-syn-A53T, which can cause iron metabolism disorder, and the increase of intracellular iron level aggravates cell senescence, which may be related to ferroptosis of nerve cells (29).

### 2.4 Mitochondrial ferroptosis

Mitochondria is the “power plants” of cellular energy, generating ATP through oxidative phosphorylation, and are the center of energy metabolism. The role of mitochondria in ferroptosis is still not well defined. However, significant changes in the morphology of mitochondria (membrane rupture and loss of cristae) occur when ferroptosis occurs, suggesting that mitochondria may play an important role in ferroptosis. When the cell undergoes ferroptosis, a variety of signaling molecules and transcription factors are activated, energy metabolism is altered, and mitochondria regulate the process of ferroptosis by adjusting the activity of the respiratory chain. When mitochondrial function is normal, mitochondria may contribute to resistance to ferroptosis; however, when mitochondrial function is impaired, the energy supply is insufficient, which may sensitize cells to ferroptosis. Since there is also a large amount of iron in the mitochondria, ferroptosis is exacerbated by an imbalance of iron metabolism in the mitochondria and an overload of iron ions. In addition, the role of mitochondria in ferroptosis involves interactions with other organelles. For example, the endoplasmic reticulum synthesizes and processes proteins and regulates calcium storage and release. During ferroptosis, alterations in the mitochondrial membrane may lead to endoplasmic reticulum stress and an imbalance in calcium ion homeostasis, which in turn triggers apoptosis. Notably, jIN’s team designed a single fluorescent probe that can label two different emissions, glutathione in mitochondria (Mito) and hypochlorous acid (HOCl) in lysosomes (Lyso). Using this dual-targeted single fluorescent probe (9-morphorino pyronine), The Mito-Lyso interaction in ferroptosis was examined (30). Thus, mitochondria co-regulate ferroptosis through interactions with other organelles.

### 2.5 Others pathways

Apart from the three classic ferroptosis mechanisms mentioned above, other new pathways have recently been identified in research. Numerous factors can impact cellular ferroptosis through the mevalonate pathway, which produces isopentenyl pyrophosphate (IPP), squalene, CoQ_10_, and cholesterol. Selenocysteine is present in the GPX4 active catalytic core, and the synthesis of selenocysteine requires the specific transporter selenocysteine tRNA, which is required for IPP and synthesis of selenocysteine tRNA. Thus inhibition of the MVA pathway will inhibit IPP levels, which in turn inhibits GPX4 synthesis, ultimately leading to ferroptosis in cells. Statins that block the mevalonate pathway can sensitize cells to ferroptosis. Moreover, there is the FSP1-CoQ_10_ pathway. Ferroptosis-suppressor-protein 1(FSP1)is a coenzyme Q (CoQ) oxidoreductase that is dependent on nicotinamide adenine dinucleotide (NADP^-^). Whereas CoQ_10_ is an antioxidant that neutralizes free radicals.FSP1 is able to reduce CoQ_10_ to panthenol (CoQ_10_-H2). Therefore, the ferroptosis inhibitory protein FSP1 blocks lipid oxidation by reducing CoQ_10_ (31).Finally, the GCH1-BH4 pathway. Tetrahydrobiopterin (BH4) is a redox-active cofactor that participates in the metabolism of NO, a neurotransmitter (32).In the production of BH4, GTP cyclohydrolase-1 (GCH1) is an essential enzyme (33).The research group of Kraft et al. identified GCH1-BH4 as a novel pathway regulating ferroptosis by CRISPR-Cas9 screening (34, 35). Overexpression of GCH1 not only eliminated lipid peroxidation, but also prevented the occurrence of ferroptosis in cells (36).

## 3 Ferroptosis plays a key role in the tumor immune microenvironment in CRC

Tumor immune microenvironment (TIME) is a complex network surrounding tumor cells, including immune cells, cancer-associated fibroblasts (CAFs), inflammatory cells, blood vessels, and extracellular matrix, which influences tumor progression and response to therapy. Metabolites produced by ferroptosis have multiple interactions with various immune cells and immune molecules in TIME and have a crucial role in tumor immunity. Ferroptosis can inhibit or promote tumorigenesis and progression. On the one hand, the release of damage-associated molecular pattern (DAMP) during cellular ferroptosi in TME can inhibit tumorigenesis; on the other hand, iron oxidative damage triggers inflammation-related immunosuppression in the tumor microenvironment can promote tumorigenesis and development. Thus, comprehending the relationship between ferroptosis and TIME could facilitate the development of novel CRC therapeutic approaches (37).

### 3.1 Immune cells and ferroptosis

#### 3.1.1 CD8^+^ T cells and ferroptosis

**Figure 2:**
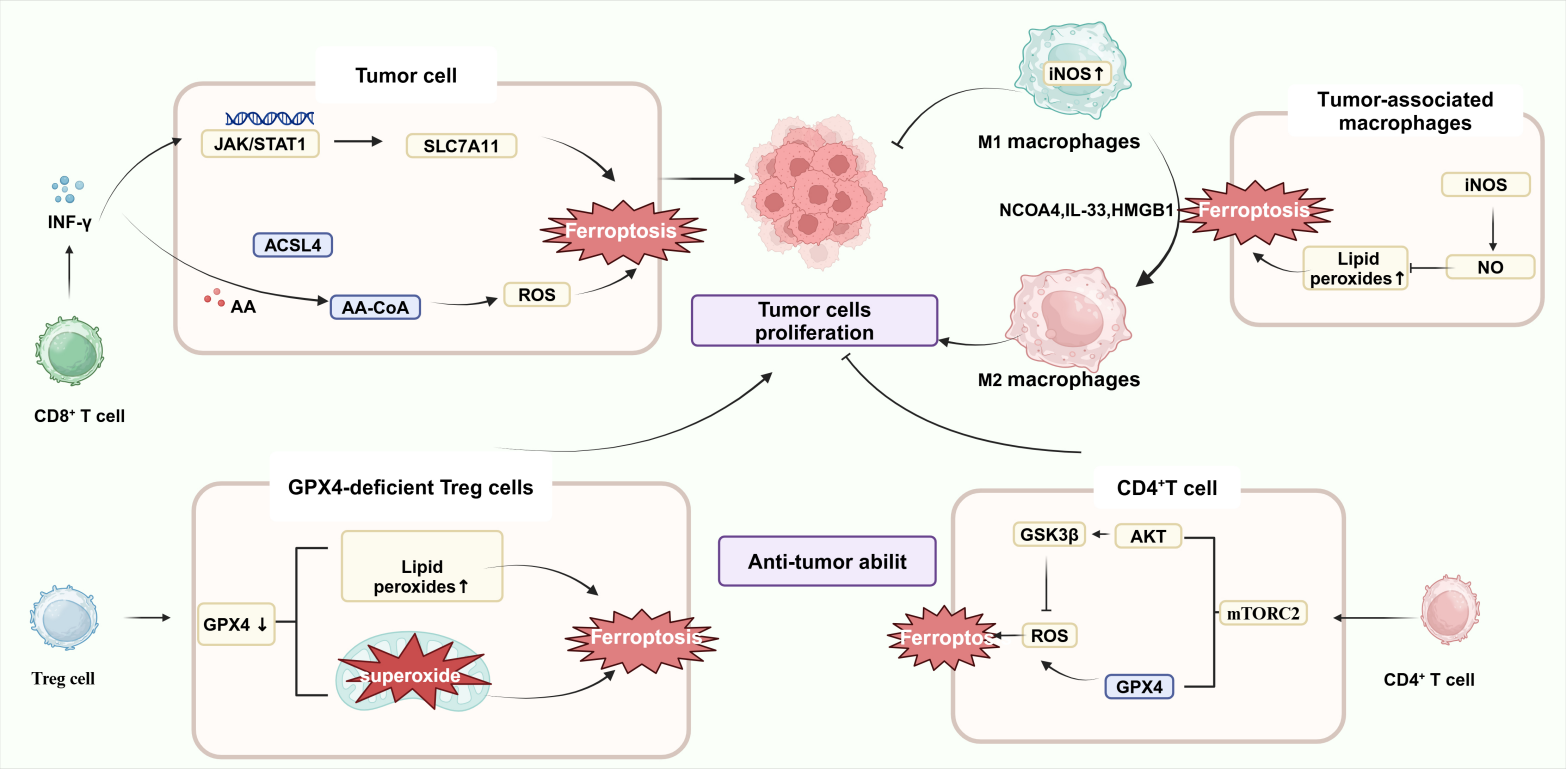
Ferroptosis and immune cells. IFN-γ, released by activated CD8^+^ T cells, down-regulated the expression of system Xc- in cancer cells, inhibited the expression of GPX4, and induced ferroptosis in cancer cells. After transfer of GPX4-deficient CD4^+^ and CD8^+^ T cells into a mouse model, both cell types underwent ferroptosis 7 days after transfer. mTORC2 mammalian target is essential for the long-term persistence of virus-specific memory CD4^+^ T cells. Mechanistically, inactivation of mTORC2 leads to impaired levels of phosphorylation of downstream AKT and GSK3β kinases, which results in aberrant accumulation of mitochondrial ROS.M1 macrophages had a higher content of NO in M1 cells due to the high content of intracellular iNOS, which can inhibit lipid peroxidation; on the contrary, M2 macrophages had a lower content of iNOS lower and produced less NO. Because of the role of iNOS, therefore, RSL3 could not induce ferroptosis in M1 macrophages but could induce ferroptosis in M2 macrophages.(Created with BioRender.com).

Improving the function of CD8^+^ T cells, one of the most deadly and toxic subsets of T cells, is essential for promoting immunological checkpoint blockade (ICB) therapy, which is a major component of antitumor immunotherapy. Notably, IFN-γ is a key cytokine in the immune-mediated ferroptosis pathway. IFN-γ released by CD8^+^ T cells down-regulates the expression of two subunits of system Xc-, SLC3A2 and SLC7A11, inhibits the uptake of cystine by tumor cells, enhances lipid peroxidation, causes ferroptosis of tumor cells, and releases DAMP (**Figure 2**). This is due to the ability of IFN-γ to activate the JAK-STAT1 signaling pathway in tumor cells, enhance the binding of STAT1 to the SLC7A11 transcription start site, and down-regulate the expression of Xc- system. DAMP further promotes the infiltration of CD8^+^ T cells in tumors, which can improve the anti-tumor efficacy of immunotherapy (38). In addition, IFN-γ can also synergize with AA to induce ferroptosis of immunogenic tumor cells in an ACSL4-dependent mechanism (39) Combined with ferroptosis inhibitors and immunotherapy with PD1 could further inhibit tumor growth (40). However, IFN-γ alone did not significantly induce cellular ferroptosis *in vitro*, suggesting a more complex regulatory network that may be related to the immune and metabolic microenvironment of the tumor. Cystathionine proteases can act synergistically with checkpoint blockade to induce potent antitumor immunity by inducing ferroptosis. Lv Y et al. demonstrated that tumor ferroptosis status (consisting of GPX4, NOX1, and ACSL4) can reflect enhanced CD8^+^ T cell infiltration based on CRC specimens (34). Later, this group discovered that APOL3-mediated ferroptosis in CRC improved CD8^+^ T cell effector activity within the tumor and increased the cells’ capacity to oppose cancer (41). In conclusion, these studies have emphasized the CD8^+^ T cell promoting tumor cell ferroptosis, suggesting that targeting the Xc-/GSH/GPX4 pathway in combination with anti-PD-1/PD-L1 antibody may be an effective tumor immunotherapy combination strategy. Not only do tumor cells exhibit ferroptosis, but T lymphocytes that infiltrate the immunosuppressive tumor microenvironment also exhibit it. Recently, Cui Guoliang’s research group team revealed a new mechanism of tumor immunosuppression. TME produces a large amount of oxidized low-density lipoprotein (OxLDL), which is taken up by CD8^+^ depleted T cells with high expression of CD36 (fatty acid transporter molecule), and the up-regulation of intracellular lipid peroxidation triggers the activation of stress protein p38 and T cell ferroptosis, which leads to the dysfunction of CD8^+^ T cell function and decrease of anti-tumor effect by the decrease of expression of TNF-α (37).

#### 3.1.2 CD4^+^ T cells and ferroptosis

Previous studies have shown that after transfer of GPX4-deficient CD4^+^ and CD8^+^ T cells into a mouse model, both types of cells are rapidly lost and undergo ferroptosis at 7 days post-transfer, suggesting that the survival and proliferation of CD4^+^ and CD8^+^ T lymphocytes are significantly influenced by GPX4 (42). In a way, this also suggests that treating CRC with inhibitors of GPX4 is likely to affect T-cell immunotoxicity. Follicular helper T cells (TFH) are a specialized subset of CD4^+^ T cells that can participate in germinal center formation and B-cell differentiation. GPX4-deficient T cells have been reported to selectively destroy TFH cells and germinal center responses in immunized mice. In addition, selenium supplementation—a trace element required for GPX4 synthesis-increases T cell GPX4 expression, boosts TFH cell counts, and encourages the germinal center response. In addition, Wang et al. found that the discovery of mammalian targets of the serine/threonine kinase complex rapamycin complex 2 (mTORC2) is critical for the long-term persistence of virus-specific memory CD4^+^ T cells. This is due to the impaired phosphorylation of downstream AKT and GSK3β kinases caused by mTORC2 inactivation, which induces abnormal accumulation of mitochondrial ROS. In addition, this signal can also down-regulate the expression of Nrf2 and inhibit GPX4 expression (43). Besides, the stimulator of interferon genes (STING) is able to recruit NK cells, T cells, macrophages and other immune cells. STING1 stimulates type I IFN responses through activation of TANK-binding kinase 1 (TBK1), leading to activation of the transcription factor IRF3.STING1 induces the activation of autophagy or immunocoagulation through mechanisms independent of TBK1-IRF3 signaling. Thus, STING1 is a multifunctional regulator of various types of RCD, including apoptosis, necrosis, pyrophosphorylation, and ferritinase-promoted, with roles in reimmunization responses. Mutian Jia et al. discovered that STING activation requires the cellular redox balance that GPX4 maintains, and that GPX4-deficient cells have enhanced intracellular lipid peroxidation, which leads to the carbonylation of STING at the C88 locus and the inhibition of its transport from the endoplasmic reticulum (ER) to the Golgi complex, thus specifically inhibiting the cGAS-STING pathway, and affecting the release of various immune cells (44).

#### 3.1.3 Regulatory T cells and ferroptosis

Regulatory T cells (Treg) are a class of immunoregulatory cells with the ability to induce immune tolerance. By controlling and coordinating the immune responses of effector T cells, mast cells, dendritic cells, and B cells *in vivo*, Treg can maintain the balance between immune cells, down-regulate the body’s response to foreign or self-antigens, and promote the progression of CRC. A poor prognosis and short survival rate are frequently linked to an increased number of Treg cells in intratumoral cells in cancer patients. PD-L1^+^ Treg Suppresses Effector T Cells and Predicts Therapeutic Effect of ICIs (45, 46). It is noteworthy that lipid perxides in GPX4-deficient Tregs cells with accumulate lipid peroxides accumulate abnormally and lead to ferrortopsis, impairing Tregs’ intra-tumoral survival, increasing T-cell infiltration into tumors, and enhancing anti-tumor immune responses, suggesting that GPX4 sustains the activation and immunosuppressive function of Tregs in TME to promote tumor immune evasion (46).Furthermore, GPX4-deficient Treg cells increased mitochondrial superoxide production and promoted IL-1β production. GPX4-specific knockdown of Treg cells inhibited tumor growth and prevented tumor immune escape without inducing significant autoimmunity. Besides, in the mouse transient middle cerebral artery occlusion (tMCAO) model, Treg cell deletion inhibits astrocyte activation via IL-10/GPX4 (47).

#### 3.1.4 B cells and ferroptosis

Different B cells have different sensitivities to ferroptosis. Different levels of CD36 in B cell subtypes could explain the differences in sensitivity to ferroptosis. B1 and marginal zone (MZ) B cells are more susceptible to lipid peroxidation than follicular (FO) B cells. In addition, defects in GPX4 in B1 and MZ B cells lead to ferroptosis and impair B cell immune function. However, GPX4-deficient FOB cells are less susceptible to ferroptosis due to lower intracellular fatty acid levels (48).

#### 3.1.5 Tumor-associated macrophages and ferroptosis

One important element affecting the effectiveness of tumor immunotherapy is TAMs (49). That is, macrophages are able to induce ferroptosis through multiple pathways, and ferroptosis products can in turn modulate TAM polarization (50, 51). It has been shown that macrophages exhibit different sensitivities to ferroptosis inducers. M1-type macrophages with high expression of iNOS were immune to ferroptosis; however, M2-type macrophages were relatively sensitive to ferroptosis. NO was able to inhibit 15-lipoxygenase-catalyzed peroxidation of polyunsaturated fatty acids in M1-type macrophages, thereby preventing ferroptosis in M1-type macrophages. Therefore, the ferroptosis inducer (GXP4 inhibitor RSL3) could not induce ferroptosis in M1 macrophages with high expression of iNOS but could kill tumor-promoting M2 macrophages, thus eliminating their immunosuppressive effects and exerting synergistic antitumor effects (52). It has been shown that THP-induced macrophages co-cultured with cigarette smoke extracts induced ferroptosis in human bronchial epithelial cells by elevated intracellular NCOA4 expression, increased ferritin degradation and up-regulation of intracellular iron levels. In addition, increased NCOA4 also increased the macrophage ratio of M2/M1. However, the mechanism of macrophage polarization induced by NCOA4 has not been clarified. However, among the proteins found in the experimental bronchial lavage fluid, IL-33 promotes M2 polarization, and HMGB-1 promotes M2 polarization. However, among the proteins found in the experimental bronchial lavage fluid, IL-33 promoted M2 polarization and HMGB-1 promoted M1 polarization, and perhaps other molecules may be involved in macrophage polarization (53).Therefore, inducer M2 macrophages undergo ferroptosis and inhibit M1 macrophage ferroptosis, which provide a novel strategy for CRC treatment.

#### 3.1.6 Dendritic cells and ferroptosis

Dendritic cells (DCs) are the most powerful specialized antigen-presenting cells (APCs) in the organism. Efimova and colleagues used animal experiments to confirm that the GPX4 inhibitor RSL3 induced cell death in mouse fibrosarcoma MCA205 cells through ferroptosis, but not apoptosis and necrosis. Furthermore, comparing the effect of treatment of MCA205 cells with RSL3 for 1 h (early tumor cell ferroptosis) or 24 h (late tumor cell ferroptosis) on the maturation of mouse bone marrow-derived dendritic cells (BMDCs), early (but not late) tumor cell ferroptosis was able to induce BMDC maturation, whereas late ferroptosis (but not early) tumor cells were mediated by BMDC phagocytosis elimination (54). Because of the low expression of SLC7A11 in DCs, the GXP4 inhibitor RSL3 selectively induced ferroptosis in DCs, whereas the SLC7A11 inhibitor erastin did not. Meanwhile, it was demonstrated that par-mediated DC ferroptosis limited anti-tumor immunity in mice by a DC vaccine model based on immunogenic cell death (55).

#### 3.1.7 Other immune cells and ferroptosis

Pathologically activated neutrophils (PMNs) in myeloid-derived immunosuppressive cells (PMN-MDSCs) are the main negative regulators of antitumor immunity. It was shown that PMN-MDSCs cells are mostly enriched in the ferroptosis pathway and that neutrophils have a higher sensitivity to ferroptosis. Ferroptosis inducers induce atypical ferroptosis in eosinophils (morphologic features consistent with ferroptosis, dependent on iron but unaffected by lipid peroxidation) and act synergistically with glucocorticoids (GCs) in the treatment of allergic diseases. In addition, there are also data suggesting that neutral ceramidase (N-acylsphingosine amidohydrolase [ASAH2]) destabilizes p53 protein to inhibit the p53 pathway in myeloid-derived suppressor cells (MDSCs) in the tumor microenvironment, thereby protecting MDSCs from ferroptosis. L-KYN produced by gastric cancer cells induced ferroptosis in Natural killer (NK) cells and NK cells with high GPX4 expression were able to resist L-KYN-induced ferroptosis. Induction of cellular ferroptosis can effectively kill tumor cells but may also impair anti-tumor immunity. In conclusion, the key molecules and signaling pathways that mediate the sensitivity of different immune cells to ferroptosis may possibly differ, offering the possibility of targeted therapy for ferroptosis or even the use of ferroptosis to kill immunosuppressed cells. In addition, combining anti-tumor immune cell modifications against ferroptosis with ferroptosis therapy may be a viable approach.

The tumor microenvironment (TME), i.e., the internal environment in which tumor cells arise and live, is a dynamic, complex and integrated system consisting of cancer cells themselves, stromal cells, different subpopulations of immune cells, the blood and lymphatic vascular system, and non-cellular components such as cytokines. During tumor development, the tumor microenvironment is not a ‘silent bystander’ but an ‘active promoter of cancer progression’. Dying cancer cells communicate with immune cells through a variety of signals during ferroptosis, thereby regulating anti-tumor immune responses. At the same time, mediators released by immune cells play a crucial role in regulating the susceptibility of cancer cells to ferroptosis. In conclusion, the interaction between ferroptosis and CRC immune cells is intricate, and ferroptosis is a double-edged sword. First, when intracellular ferroptosis occurs leading to cellular damage, DAMP (e.g. ATP,HMGB1, etc.) is released and acquires immunogenicity, and is able to bind to pattern recognition receptors, such as Toll-like receptor 4 (TLR4) and purinergic receptor P2X7, to promote activation of inflammation and the innate immune system, and to enhance the anti-tumor through activation of immune system immune effects. There is also STING mentioned above, which promotes tumor immunity by activating the cGAS/STING signaling pathway and releasing IFN-β.The IL-33 and HMGB1 (which are DAMPS) mentioned above that modulate the macrophage program are then able to ferroptosis the tumor immunity of dead cells. In addition, a study has shown that HMGB1 can promote erastin-induced ferroptosis by up-regulating cyclooxygenase 2 and transferrin expression via the RAS-JNK/p38 pathway. In addition, ferroptosis that induces the release of CRT and ATP from DAMPs can promote the maturation and recruitment of antigen-presenting cells. Second, various immune cells secrete substances that modulate ferroptosis of tumor cells. So when targeting ferroptosis to treat tumors, it is also important to be aware of the effects on immune cells. The use of ferroptosis inducers is capable of killing tumor cells, but at the same time may inhibit the activity and survival of other anti-tumor immune cells (56). Hematopoietic cell signaling Transducer (HCST) and tyrosine kinase binding protein (TYROBP) are trigger receptors expressed on bone marrow cells 2 (TREM2) and play a key role in the immune response to disease. Studies have shown that TREM2, HCST and TYROBP can participate in NK cell-mediated cytotoxicity and b cell receptor signaling pathways, and are positively correlated with most immunomodulators (57). However, the interaction between TREM2, HCST, TYROBP and ferroptosis remains to be investigated. With the present studies, the evidence that ferroptosis promotes anti-tumor immunity is much stronger, meaning that the anti-tumor effect of ferroptosis is greater than the promotional effect. Moreover, most of the existing studies have focused on the interaction of individual immune cells with ferroptosis, and few studies have addressed the role of the overall body immune system with ferroptosis, which still needs further elucidation.

### 3.2 CAFs and ferroptosis

CAFs are an important part of the tumor microenvironment and are the main stromal cells in the TME, which can promote tumor cell proliferation, differentiation and metastasis through the secretion of various types of cytokines, and can even promote tumor progression by regulating tumor angiogenesis and immune system suppression. Recently many studies have shown that CAFs regulate ferroptosis in tumor cells. CAFs-derived exosome miR-522 inhibits arachidonic acid lipoxygenase 15 (ALOX15) and reduces cytoplasmic lipid ROS accumulation. In addition, CAF can affect ferroptosis in other immune cells. For example, CAF promotes ferritin autophagy in NK cells and increases intracellular iron ion overload, which is necessary for ferroptosis. Interestingly, however, CD8^+^ T cells have been found to counteract the antioxidant strategy of CAFs.IFN-γ produced by CD8^+^ T cells alters the metabolism of cystine and GSH in the cells of CAFs, leading to reduced intracellular levels of cystine and GSH, which are sensitive to ferroptosis.

### 3.3 The extracellular matrix and ferroptosis

The extracellular matrix is a highly active part of the tumor microenvironment and influences tumor biological progression. Epithelial-mesenchymal transition (EMT) plays an important role in tumor, and ECM can greatly affect the EMT process. In addition to proteins and micrornas can affect the process of EMT, the influence of circular RNA on the process of EMT cannot be ignored. The onco-suppressor circRNAs inhibit EMT, while the tumor-promoting circRNAs mediate EMT for acceleration of carcinogenesis (58). Nanoparticles induce ferroptosis in endothelial cells, inducing autophagic degradation of ferritin, and that this is dependent on the autophagic pathway. Mechanistically impaired mitochondrial function and increased reactive oxygen species activate the AMPK-ULK1 axis, which subsequently activates ferritin autophagy, leading to an increase in intracellular ferric ions, which leads to subsequent lipid peroxidation, resulting in cellular ferroptosis.

## 4 Ferroptosis in CRC

### 4.1 Factors that influence the susceptibility of CRC cells to ferroptosis

#### 4.1.1 Factors that make CRC cells sensitive to ferroptosis

CRC is more strongly associated with ferroptosis than other cancers. First, due to the special environment of the gastrointestinal tract, TFRC is overexpressed in CRC in colorectal cancer, and second, colorectal cancer is the only malignancy that maintains a source of iron uptake, which leads to the presence of excess iron in cells, and ferroptosis is more likely to occur (59).In addition, the ROS content in CRC cells is usually higher than that in other normal cells. Thus ROS may induce ferroptosis in cancer cells without affecting normal cells. However, the accumulation of ROS is not always beneficial to cells. Moderate levels of ROS can lead to cell damage, DNA mutation and inflammation, and too high ROS can cause cell death (60, 61).

#### 4.1.2 Factors that make CRC cells less sensitive to ferroptosis

But in reality, CRC cells also have this ferroptosis defense activation, and are not as susceptible ferroptosis as they should be. As one of the most important regulators of ferroptosis, GPX4 is highly expressed in CRC, and the high expression of GPX4 is associated with poor prognosis of CRC (62). In summary, the regulatory system of ferroptosis in CRC cells remains to be explored. Targeting ferroptosis-resistant factors may be an effective way to treat CRC.

### 4.2 Sensitivity of different subtypes of CRC cells to ferroptosis

It is an effective therapeutic strategy to regulate the biological process of CRC cells by ferroptosis. Different types of CRC are treated in different ways, and more targeted treatment of CRC may have unexpected therapeutic effects. We speculated that colorectal cancer cells may also have different sensitivity to ferroptosis. Currently, four consensus molecular subtypes (CMSs) are internationally recognized, namely CMS1,CMS2,CMS3, and CMS4. Li et al. used biological information to study the TME characteristics of different CMS in CRC, and the authors found that the expression of genes related to ferroptosis varied greatly among different CMS. Among them, ferroptosis was significantly inhibited in CMS3, but relatively active in CMS1 and CMS4. In addition, CD8^+^T cells, TFH, and M1 macrophages had higher infiltration levels in CMS1, while Treg cells and monocytes had decreased infiltration levels. CMS4 is dominated by M0 and M2 macrophages, with few M1 macrophages and CD8^+^T cells (63). But the conclusion is just based on the analysis of TCGA RNA - seq, still need further clinical test and verify. The CMS mentioned above are grouped based on transcriptomics, while CRCS can also be divided into APC,KRAS,BRAF, TP53 and others based on genomics. At present, KRAS mutant CRC has been studied more. Inactivation of the APC gene is associated with activation of the Wnt/β-catenin signaling pathway and may affect ferroptosis sensitivity.TP53 mutation may regulate ferroptosis by affecting SLC7A11 expression (64).KRAS mutations are associated with upregulated expression of FSP1, which protects cells from ferroptosis (65). KRAS may also maintain high iron levels required for CRC growth by regulating HIF-2α and JAK/STAT pathway signaling, and JAK promotes tumor growth through iron input mediated by DMT1 (23, 24). It has also been shown that KRAS can regulate glutamine hydrolysis, mitochondrial ROS production and glucose uptake in CRC, all three of which have indirect effects on ferroptosis (66). In addition, recent studies have shown that KRAS mutant tumors in male CRC patients are altered in terms of GSH biosynthesis, TSP activity, and methionine metabolism. High expression of GPX4, FTH1, and FTL, as well as low expression of ACSL4, were also associated with poorer 5-year OS in men with KRAS mutant tumors (67). Bromelain effectively induces ferroptosis in Kras mutant colorectal cancer cells and plays a cytotoxic role (68). Cetuximab enhanced the cytotoxic effect of RSL3 on KRAS mutant CRC cells (69). Therefore, using the characteristics of different types of CRC, targeted ferroptosis therapy may be a potential precision medicine strategy.

## 5 Ferroptosis-related indicators contributed to early diagnosis and prognosis of CRC

CRC is one of the few malignant diseases for which precancerous lesions or early-stage tumors can be detected using appropriate screening methods, thereby reducing morbidity and mortality through appropriate clinical interventions. Colonoscopy is the recognized gold standard for screening, but the inconvenience of the procedure and the risk of infection through perforation of the bowel prevent it from becoming a mass screening tool. The fecal occult blood test (FOBT) is inexpensive and can reduce CRC mortality by identifying cancerous lesions, but has a limited role in reducing CRC incidence. Several blood biomarkers relevant to CRC surveillance and prognostic assessment are currently used in many clinical laboratories.

Ferroptosis-related genes have been shown in numerous studies to potentially aid in the early detection of CRC. Metallothionein-1G (MG1T), a ferroptosis-associated gene, is a member of metallothioneins (MTs) involved in the oxidation and regulation of metalloproteinases (70). MT1G was previously reported to inhibit ferroptosis by blocking glutathione depletion-mediated lipid peroxidation in hepatocellular carcinoma cells and clear cell renal cell carcinoma, and to promote sorafenib resistance in hepatocellular carcinoma cells. Recently, it has also been shown that CRC patients with low MT1G levels have a poorer prognosis. In addition, MT1G expression is closely related to the immune microenvironment and is involved in tumor progression. Therefore, MT1G may serve as a potential prognostic biomarker and immunomodulatory factor for CRC (71). In addition, a recent study by Demir et al. showed that ferritin is an effective marker for predicting the prognosis of CRC and that high levels of hemoferritin may indicate poor survival (72). Transferrin is a glycoprotein that plays a role in iron absorption and iron ion transport. Sheng et al. showed that transferrin testing is more accurate than immune fecal occult blood testing in detecting CRC and precancerous lesions (73). In recent years, many studies have been devoted to screening differentially expressed genes for ferroptosis in CRC and establishing predictive models. Shao et al. constructed a risk assessment model to predict the prognosis of CRC patients using ten genes associated with ferroptosis in CRC. The reliability of this model was validated in multiple datasets and clinical specimens. Importantly, there was also a significant difference in immune infiltrating cells between the high- and low-risk groups in this model, further validating the interaction between ferroptosis and TIME (74). One of the main components of the enhanced unfolded protein response (UPR), which binds to target proteins to keep them stable, is HSPA5.HSPA5 enhances cell proliferation and chemoresistance and promotes CRC progression (75). Recent studies have shown that HSPA5 interacts with and maintains the stability of GPX4 and inhibits ferroptosis in CRC cells, providing a new target for the prognosis and treatment of CRC patients (76). Recent studies have also shown that non-coding RNAs can regulate ferroptosis through multiple pathways and are expected to be new therapeutic targets and early diagnostic markers for a variety of cancers, including CRC (77).

The above proteins or models can provide some value for the diagnosis and prognosis of CRC and may be an auxiliary index for clinical judgment of CRC.

## 6 Application prospect of ferroptosis in therapy of CRC

Clinical treatments for CRC mainly include surgery, chemotherapy and immunotherapy. Multiple genes and signaling pathways are involved in the intricate process of tumor chemoresistance. The emergence of tumor chemotherapy resistance is one of the most important causes of tumor treatment failure, which is a great challenge for clinical treatment. Therefore, the five-year survival rate of patients remains low due to tumor recurrence, metastasis, and the resistance of CRC cells to chemotherapeutic treatments. With the growing interest in ferroptosis, many scholars many attempts to target ferroptosis as a new strategy for clinical treatment, combining conventional treatment options with ferroptosis, such as alone, in combination, or with targeted therapy, may have unexpected results targeting drugs may have unintended effects.

### 6.1 The use of ferroptosis in chemotherapy of CRC

Ferroptosis is a new mechanism for inducing cancer cells to undergo death in recent years, and researchers are constantly investigating drugs based on ferroptosis in tumor cells. The occurrence of ferroptosis is closely related to many biological processes in the body, such as glutathione, iron, and polyunsaturated fatty acid metabolism. Giving appropriate intervention to these metabolic processes may modulate the sensitivity of tumor cells to ferroptosis. It has been shown that cisplatin, as a first-line anticancer drug in CRC patients, induces ferroptosis in CRC cells HCT116 (78, 79). The anticancer drug Paclitaxel modulates lipid peroxidation by Park 3 and SLC7A11 to induce ferroptosis in CRC cells. CRC cells may inhibit cellular ferroptosis through the KIF20A/NUAK1/PP1β/GPX4 pathway, and this may also contribute to the resistance of CRC to oxaliplatin. Cysteine desulfurase (NFS1) deficiency synergistically with oxaliplatin enhances the sensitivity of CRC cells to oxaliplatin by triggering PANoptosis (apoptosis, necrosis, pyroptosis, and ferroptosis) through increased cellular ROS levels. In addition, the combination of cisplatin with erastin enhances its antitumor effects. By forming complexes with human and bacterial iron transporters, lipocalin 2 (LCN2) inhibits bacterial growth and maintains iron homeostasis. Since LCN2 inhibits ferroptosis in CRC cells by decreasing intracellular iron levels and stimulating the expression of GPX4 and xCT, elevated levels of LCN2 are associated with tumor progression and drug resistance. Inhibition of LCN2 function using LCN2 monoclonal antibody reduces *in vitro* chemoresistance and transformation, and reduces tumor progression and chemoresistance in xenograft mouse models. Thus LCN2 may be an important therapeutic strategy for resistance to conventional chemotherapy in CRC (80). Cetuximab is the standard treatment for KRAS wild-type mCRC, but has limited efficacy against KRAS-mutant CRC cells. Studies have shown that cetuximab not only activates p38 MAPK to inhibit the Nrf2/HO-1 axis to enhance RSL3-induced ferroptosis, but also enhances the cytotoxic effect of RSL3 on KRAS-mutant CRC cells, which may be promising to help develop attractive therapeutic strategies for patients with KRAS-mutant CRC (81). Furthermore, ACSL4 is expressed more in human CRC cells with Kras mutants, and bromelain can increase ACSL4 expression in cells, cause ferroptosis in CRC cells, and inhibit the growth of tumors (68). This suggests that the Kras gene may be an upstream regulator of ferroptosis, which induces ferroptosis in cells by promoting ACSL4 expression.

### 6.2 The role of ferroptosis in immunotherapy of CRC

Cancer immunotherapy produces anti-tumor effects by enhancing anti-tumor immune response and relieving tumor immunosuppression. Immune checkpoints are molecules that are expressed on immune cells and regulate the degree of immune activation. Normally, immune checkpoints (ICs) are involved in the negative regulation of the immune response to avoid autoimmune response damage. Thus tumor cells produce immune evasion by lowering the immune system and promote malignant tumor progression (82, 83). However, some patients show low sensitivity and resistance to ICI therapy, which may be related to a variety of factors such as host intrinsic and environmental factors. Many studies now attempt to combine ICI therapy with other treatments to improve the efficiency of ICI. In tumors, ferroptosis and immune checkpoints interact through TIME to achieve homeostasis in the complex TME and maintain and promote tumor growth. By promoting or blocking the substances or pathways that interact with the two, the original “homeostasis” changes from promoting tumor growth to inhibiting tumor growth.

As mentioned above, IFN-γ secreted by CD8^+^ T cells can downregulate System Xc-, lipid peroxidation in tumor cells, leading to ferroptosis. Blocking PD-1 inhibitors in CRC cells has been reported to block IFN-γ, enhance the abundance of CD8^+^ T cells, and ultimately activate the immune system to attack cancer cells (84). However, under certain circumstances, INF-γ was able to induce tumor cells to undergo immune escape, and INF-γ secreted by CD8^+^ T cells in ovarian cancer cells was able to promote the high expression of PD-L1, which helped the tumor cells to undergo immunity, and promoted tumor progression. Therefore, IFN-γ signaling plays multiple roles during ICI therapy and is at the intersection of ICI therapy and ferroptosis.

First, immunotherapy can be used in combination with ferroptosis inducers. Previous studies have found greater tumor growth inhibition in mice treated with a combination of PD-L1 blocker or Cystine enzyme compared to mice treated with PD-L1 blocker or Cystine enzyme alone (38). Notably, increased expression of TYR03 was found in anti-PD-1 resistant tumors. The researchers then found that a TYR03 receptor tyrosine kinase (RTK) inhibitor, which promotes ferroptosis and sensitizes tumors to anti-PD-1 therapy. This is because the TYR03 signaling pathway upregulates the expression of key ferroptosis genes such as SLC3A2, which inhibits tumorigenic ferroptosis (85). It is noteworthy that CD8^+^ T cells are more sensitive to ferroptosis than cancer cells and are susceptible to spontaneous ferroptosis by TME, resulting in CD8^+^ T cell death and promoting immune evasion by cancer cells. Activated CD8^+^ T cells were also more sensitive to rsl3-induced ferroptosis than cancer cells. ACSL4-deficient CD8^+^ T cells were protected from the threat of rsl3-induced ferroptosis but also resulted in functional defects of CD8^+^ T cells, allowing cancer cells to evade specific killing by CD8^+^ T cells. Thus, ACSL4, a key enzyme that regulates lipid peroxidation during cellular ferroptosis, is essential for maintaining CD8^+^ T cell function. Furthermore, zero-valent iron nanoparticles (ZVI-NP), the ferroptosis inducer, induced cancer-specific cytotoxicity reducing the number of Tregs and decreasing the expression of PD-L1 on tumor cells and PD-1/CTLA4 on CD8^+^ T-cells, exerting an anti-tumor immune effect (86). Recent studies have shown that RSL-3-enriched nanoparticles promote immune death of tumor cells, and blocking PD-L1 combination therapy further enhances T-cell infiltration in the TME (87).

In addition, deferroptosis sensitization of anti-tumor immune cells followed by combined ferroptosis therapy might be an effective way to treat CRC. In other words, it is the combination of immunotherapy with ferroptosis inhibitors in TIME that inhibits ferroptosis in immune cells and promotes tumor immunity in the body. Inhibition of CD36 expression in CD8^+^ T cells protects cells from ferroptosis and improves the effectiveness of ICI immunotherapy. In addition, Ferrstatin-1, an inhibitor of ferrortopsis, stimulates IFN-γ production and CD8^+^ T cell proliferation and can be used in combination with immunotherapy (40). The combination of Ferostatin-1 with an immune checkpoint inhibitor significantly reduced tumor growth in mice *in vivo*, whereas no significant benefit was observed with either inhibitor alone (83).

### 6.3 The role of ferroptosis in the herbal treatment of CRC

Chinese medicines can reduce the toxic side effects of radiotherapy, and some Chinese medicines combined with chemotherapy or radiotherapy can enhance the immunity of CRC patients, improve the body’s tolerance to chemotherapy and radiotherapy, and reduce the side effects and complications caused by chemotherapy and radiotherapy. At present, the anticancer activity of many Chinese herbal medicine extracts or active monomer compounds has been confirmed. A new interpretation of the anti-tumor mechanism of traditional Chinese medicine from the perspective of ferroptosis, which provides a new way of thinking for Chinese medicine against tumors. It has been found that ginsenoside Rh3 (GRh3) prevents the entry of Nrf2 into the nucleus, which inhibits SLC7A11, leading to depletion of GSH, which decreases GPX4 activity, ultimately leading to the accumulation of iron, ROS, and malondialdehyde (MDA), and ferroptosis in CRC cells (88).β-elemene, an active substance isolated from turmeric, can inhibit KRAS mutant CRC tumor growth by inducing ferroptosis combination with cetuximab and inhibit tumor migration by modulating EMT. This is expected to provide a prospective therapeutic strategy for the treatment of KARS mutant metastatic colorectal cancer (mCRC) patients (89).The natural product β-Lapachone promotes lipid peroxidation by reducing GSH synthesis through the xCT/GPX4 axis, in addition it promotes ferroptosis in CRC cells through NCOA4-mediated ferritin autophagy (90).The current study by our group also demonstrated that the herbal extract 2’,4’-dihydroxychalcone can affect the proliferation, migration and ferroptosis process of CRC cells through c-myc/CLIC3. Studies on the mechanism of action of traditional Chinese medicine have revealed that Auriculasin, Glycyrrhetinic acid and Shuganning injection can also assist in tumor therapy by regulating ferroptosis (91–93). In addition, recent studies have shown that baicalein, triptonide and emodin can target JAK2/STAT3/GPX4,SLC7A11/GPX4 and NCOA4/FTH1 signaling axes to promote ferroptosis in CRC cells, respectively, which provide more possibilities for clinical treatment (94–96). Cisplatin can promote the activation of the antioxidant system Nrf2/HO-1, while ginkgetin can inhibit the antioxidant system Nrf2/HO-1, and the combination of the two can promote the anti-tumor effect of cisplatin in non-small cell lung cancer cells (97). Ginsenoside CK (CK) affected ferroptosis of HepG2 and SK-Hep-1 cells and inhibited cell proliferation by influencing FOXO1 (98). All of these herbs may be used in the treatment of CRC. This provides a new research direction for the clinical treatment of CRC.

In addition, as a novel tumor therapeutic drug, the ferroptosis nano-formulation exerts anti-tumor therapeutic effects by exploiting the redox imbalance caused by the excessive accumulation of lipid peroxides, which triggers the death of CRC cells. According to the characteristics of the occurrence of ferroptosis in different tumors, the nano-formulations that can be used for ferroptosis therapy and the combination therapy with traditional therapies hold great promise.

### 6.4 Emerging technologies and ferroptosis treatment in CRC

Existing small molecule ferroptosis inducers have limitations such as poor water solubility, drug resistance, and low targeting, which hinder their clinical application. Introducing ferroptosis inducers or inhibitors into the tumor microenvironment through nano-delivery system to promote the occurrence of ferroptosis in tumor cells has become the CRC tumor immunotherapy. A new nanomedicine (RCH NPs), co-assembling hemoglobin (triggered ferroptosis), celecoxib (disrupts inflammation-associated immunosuppression) and roscovitine (inhibits PD-L1 expression) with the help of human serum albumin, induced cellular ferroptosis and at the same time was able to inhibit ferroptosis-induced immunosuppression. In addition calcium/manganese hybridized bifunctional nanomaterials (CMS), which act as both ferroptosis inducers and immune adjuvants in triple-negative breast cancer (TNBC), enhance tumor immunogenicity and reverse the immunosuppressive microenvironment by inducing ferroptosis and activating innate immunity. Graphene oxide(a nanomaterial) has unique physicochemical properties and has great potential in the diagnosis and treatment of CRC. It is beneficial to use the GO delivery device to deliver drugs to induce ferroptosis in CRC cells (99).Thus, nanomaterials have shown superior therapeutic effects in tumors.

## 7 Side effects of antitumor therapy targeting ferroptosis

Tumor cell metabolism is disturbed, redox homeostasis is destroyed, and it is susceptible to induction of ferroptosis. Targeting ferroptosis is important for many diseases such as cancer, but there are still many problems in ferroptosis treatment.

### 7.1 Immune cell death

As mentioned above, the role of tumor cells and immune cells is complicated, and ferroptosis inducers may “kill” anti-tumor immune cells while inducing tumor cell death. In DC2.4 cells, the ferroptosis inducer RSL3 can activate the PPARG/PPARγ pathway involved in lipid metabolism to induce ferroptosis, thereby impairs DC maturation and limits anti-tumor immunity (100). In addition, GPX4-deficient Treg cells were susceptible to ferrpptosis and showed elevated IL-1B production, which in turn enhanced the T Th17 (helper cell 17) response in B6 mice vaccinated with B16.F10 melanoma cells (46). In contrast, overexpression of GPX4 or depletion of CD36 can save the effective death of cytotoxic CD8^+^ T cells and enhance their anti-tumor ability (37). Due to the lack of cell or tissue selectivity of currently widely used ferroptosis activators, it may lead to unexpected cell death in various immune cell types, undermining antitumor immunity to cancer. The good news is that researchers have discovered the first cell-specific inducer of ferroptosis, N6F11. Tripartite motif containing 25 (TRIM25) is mainly expressed in cancer cells, but not in immune cells. N6F11 selectively induces ferroptosis in cancer cells in a variety of models by targeting TRIM25-mediated GPX4 degradation in pancreatic cells (101).

### 7.2 Organ damage

Liver and kidney are the most important organs of body metabolism, and many cancer treatment drugs are metabolized by liver and kidney. The heavy use of drugs, which can increase the exposure of these organs to potentially harmful substances, greatly increases the burden on the liver and kidneys. Similarly, similar to some other anticancer agents, specific ferroptosis inducers have the potential to induce hepatorenal toxicity. Research shows that in selective knockout GPX4 liver cells and cells in the kidney cells can occur in ferroptosis, but vitamin E can be reversed to some extent the toxic effect (102–104).What’s more, may cause stem cells and bone marrow damage. This is because healthy hematopoietic stem cells have low levels of protein synthesis and are susceptible to ferroptosis induced by compounds such as erastin, FIN56, FINO2, and RSL3 (105).

### 7.3 Secondary tumorigenesis

Secondary tumorigenesis refers to newly developed tumors after treatment of the primary tumor, and their occurrence may be related to factors such as gene mutation, immunosuppression or viral activation during treatment. Studies have shown that diets involving high iron or GPX4 deficiency promote pancreatitis and pancreatic tumor development in mice. Conversely, inhibition of ferroptosis by administration of liproxstatin 1 reduced the formation of spontaneous pancreatic cancer (50, 106).

## 8 The challenge for the targeted ferroptosis treatments

We must control the side effects of ferroptosis and the toxic effects of the ferroptosis inducer to maximize the therapeutic effect.

### 8.1 Cell or tissue specificity

High specificity and selectivity are required to minimize the potential toxicity of drugs or ferroptosis inducers. Currently, most ferroptosis inducers lack cell or tissue specificity and harm many normal cells. Therefore, it is critical to find drugs that can target ferroptosis in tumor cells, but have no or low toxicity to normal cells.

### 8.2 Drug carrier

We need advanced drug delivery systems that allow targeted controlled release of our novel molecules within tissues and cells to optimize their potential benefits to patients. The development of targeted drug delivery systems is critical to improving therapeutic outcomes and reducing systemic side effects. Nanoparticles have the advantages of enhancing drug stability, solubility and targeted delivery. Scientists aim to break down the barriers between our most promising new drug candidates and their targets in tissues and cells. Recent studies show promise for using nanoparticles, including liposomes, micelles, and polymer carriers, to address these challenges.

### 8.3 Biomarker identification

Some biomarkers of ferroptosis, such as TFRC, ACSL4, prostate-independent peroxidase synthetase 2 (PTGS2)and hyperoxidized PRDX3, are measured at the mRNA or protein level to monitor the ferroptosis response (10, 107–109). However, for blood biomarkers, which have more clinical significance, there are few studies. There is an urgent need to develop an easy-to-apply and cost-effective biomarker or method for clinical trials.

### 8.4 Clinical transformation

Accelerating the transformation of the basic research results of ferroptosis into clinical applications is crucial to achieve early diagnosis, individualized treatment and accurate prognosis.

## 9 Summary and outlook

The discovery of ferroptosis has expanded our knowledge of cell death forms. In cancer, ferroptosis is a double-edged sword that may inhibit or promote tumor growth, depending on the cell and tumor type. This review focuses on the modulation and regulation of ferroptosis in CRC and its interaction with TIME. However, while the key to inducing ferroptosis has been unearthed, it is undeniable that we have not yet identified the key execution proteins that induce cellular ferroptosis, nor can we determine whether other key pathways exist, which still need to be further explored. Since the proposal of ferroptosis, more and more studies have shown the potential of inducing ferroptosis in tumor cells for tumor therapy. Current clinical studies are mainly targeting iron metabolism, lipid metabolism, antioxidant barriers and other key pathways of ferroptosis for cancer treatment, and many ferroptosis-inducing drugs are also used for tumor therapy. Ferroptosis can be used in combination with chemotherapy, immunotherapy, and herbal therapy to enhance therapeutic efficiency. Overall, the ferroptosis regulatory network is extremely complex and highly context-dependent in CRC. Although more and more researches have revealed the mechanisms and metabolic alterations involved in ferroptosis in cancer cells, they are still at a preliminary stage, and there is a urgent need to analyze the role of ferroptosis in the cancer immune system from the perspective of metabolic pathways, epigenetic modifications, and gene mutation profiles. Finding tumor cell-immune cell balance in ferroptosis is key. Inducing ferroptosis in tumor cells without affecting the immunological activity and number of immune cells is an urgent issue to be solved. The development of ferroptosis-inducing therapies for cancer still requires further studies targeting regulatory mechanisms and signaling pathways. The search for biomarkers to facilitate the tracking of ferroptosis cells and the induction of ferroptosis *in vivo* will be an active area of research in the future. Exploring the mechanisms in which ferroptosis affects immune cells, these molecular mechanisms may be used for future disease targeting therapeutics or diagnostics, such as the development of cancer immunotherapy based on ferroptosis cell vaccines, the development of inhibitors against DAMPs and related nanomaterial drugs.
